# Steam-sterilizable cationic nanodiamond–silver composites with enhanced antibacterial activity

**DOI:** 10.1039/d6na00095a

**Published:** 2026-05-29

**Authors:** Katerina Kolarova, Hana Stiborova, Simona Lencova, Maksym Bilozerskyi, Oleksandr Romanyuk, Alexander Kromka, Stepan Stehlik

**Affiliations:** a FZU – Institute of Physics of the Czech Academy of Sciences Cukrovarnicka 10 162 00 Prague Czech Republic stehlik@fzu.cz; b Department of Biochemistry and Microbiology, University of Chemistry and Technology Technicka 5 166 28 Prague Czech Republic

## Abstract

Hydrogenated nanodiamonds (H-NDs) combine high biocompatibility with a positive *ζ*-potential, yet exhibit only weak and medium-dependent antibacterial effects. Here, we engineer H-NDs (median sizes 18 and 125 nm) into Ag-decorated composites (H-ND–Ag) without suppressing their positive surface charge (cationic character) by adsorbing a thin chitosan layer, followed by mild polyethyleneimine-assisted AgNP formation. We evaluate how steam sterilization by autoclaving affects the surface chemistry and dispersion of both pristine H-NDs and H-ND–Ag. Autoclaving perturbs the neat H-ND, leading to decreased *ζ*-potential and eventual aggregation. In contrast, the chitosan–Ag shell preserves the positive charge and long-term colloidal stability of H-ND–Ag. The H-ND–Ag composites suppress the growth of *Escherichia coli* and *Staphylococcus aureus* at low loadings, matching or exceeding the performance of reported, protein-stabilized ND–Ag benchmarks. The results support a contact-mediated “Ag-patch” delivery mechanism in which carrier size and Ag distribution – rather than surface charge alone – dominate antibacterial efficacy, and establish a protein-free, autoclave-sterilization-compatible route to durable antibacterial nanodiamond formulations.

## Abstract 2

Hydrogenated nanodiamonds exhibit limited intrinsic antibacterial activity and reduced colloidal stability after steam sterilization, but enable robust, sterilizable AgNP-based antibacterial composites.

## Graphical abstract

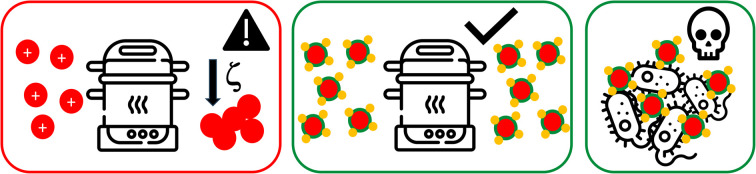

## Introduction

Nanodiamonds (NDs) are carbon nanomaterials with distinctive physico-chemical attributes: high specific surface area, tailorable surface chemistry, mechanical robustness, and biocompatibility, which enable applications spanning biomedicine, quantum technologies, catalysis, and environmental remediation.^1–4^ Their optical readouts (*e.g.*, color centers with stable fluorescence and negligible photobleaching), and chemical versatility have further catalyzed interest in ND-based “smart” platforms for imaging, sensing, and delivery.^5^

Despite this promise, reports of intrinsic antibacterial activity of unmodified NDs remain mixed and strongly context-dependent. Media composition, ionic strength, and protein corona formation modulate ND aggregation and surface charge, making a simple mechanistic explanation difficult. For example, colony reductions sometimes observed with *Staphylococci* in salt-rich media have been attributed primarily to heteroaggregation between NDs and bacteria rather than to bactericidal mechanisms, while *E. coli* often shows a negligible response under comparable conditions.^6,7^ Collectively, these observations caution that ND surface chemistry, dispersion state, and test medium are pivotal for interpreting “antibacterial” ND effects.

One robust strategy to move beyond these limitations is to use ND as a tunable carrier for catalytically or photophysically active nanoparticles. ND–Au hybrids exemplify this approach, combining plasmonics for surface-enhanced Raman spectroscopy (SERS) analysis, photoacoustic imaging, and photothermal transduction with the chemical stability and biocompatibility of NDs.^1,8–10^ In antimicrobial contexts, ND–Ag composites are particularly compelling: immobilizing Ag nanoparticles (AgNPs) on NDs can mitigate uncontrolled AgNP aggregation at bacterial surfaces and sustain contact-mediated activity.^11,12^ Chang *et al.* demonstrated that BSA-stabilized ND–Ag (Ag–ND@BSA) achieves low µg mL^−1^ minimal inhibitory concentration (MIC) values against *E*. *coli* and *S*. *aureus*, highlighting an ND-enabled “Ag-patch” delivery effect.^11^

The charge state is another important parameter. Most published ND composites employ oxidized, negatively charged NDs, yet many bacterial cell walls are net negative, suggesting that positively charged carriers could enhance initial adhesion and local dosing *via* electrostatics. For other nanoparticle families, cationic surfaces enhance electrostatic interactions with negatively charged bacterial membranes and often lead to lower MIC values,^13,14^ whereas anionic analogues tend to rely more on ROS-mediated antibacterial pathways.^15^ Positively charged hydrogenated NDs (H-NDs) are, therefore, attractive carriers: hydrogen termination yields unusual electronic/surface properties, including positive *ζ*-potential in water, while maintaining ND biocompatibility.^16^ However, the intrinsic antibacterial action of H-NDs alone is, at best, weak and medium-dependent, and appears markedly inferior to purpose-engineered cationic systems or to ND–Ag hybrids.^7,17^

Translational deployment further requires validated sterilization. Biologically used colloids must be sterile, yet sterilization can alter nano-surface chemistry and colloidal stability. Ultrafiltration can remove microbes without changing chemistry but is limited by particle size and does not address endotoxins.^18^ Gas-phase plasma and UV can oxidize carbon surfaces.^18,19^ Sterilization by autoclaving – the most practical and broadly accepted method – has been reported to modify some carbon nanomaterials while leaving others comparatively intact.^20,21^ For NDs and related carbons, autoclave conditions (∼121 °C, saturated steam) may shift surface functional groups and *ζ*-potential, with consequences for dispersion and bio-interactions.^22,23^ In H-NDs specifically, FTIR studies have linked the ∼1330 cm^−1^ Fano-type feature (interplay of the diamond phonon and near-surface charge carriers) to surface/electronic states that are sensitive to oxidation; low-temperature oxidation of hydrogenated detonation NDs has been observed starting near 100–150 °C.^24–26^ The positive *ζ*-potential often attributed to H-NDs is likewise interpreted through protonation/transfer-doping frameworks rather than idealized, perfectly C–H-terminated surfaces.^27,28^

In this context, two pragmatic questions motivate this work. Can positively charged H-NDs act as efficient carriers for AgNPs, potentially using electrostatic adhesion, when compared to the more common protein-stabilized, negatively charged ND–Ag constructs?^17^ And how does steam sterilization by autoclaving modify the H-ND surface chemistry, *ζ*-potential, and dispersion state, and can a composite architecture buffer these changes without sacrificing antibacterial function?

To address these issues, we prepare hydrogenated HPHT NDs with median sizes of 18 and 125 nm (H-ND18 and H-ND125) and positive zeta potential, convert them into still cationic H-ND–Ag composites (H-ND18–Ag and H-ND125–Ag) *via* a minimal chitosan shell and mild polyethyleneimine (PEI) reduction, and compare neat H-NDs and H-ND–Ag before and after autoclaving. We correlate DLS/*ζ*-potential and FTIR data, including the evolution of C–H and O–H/C=O bands and the ∼1330 cm^−1^ Fano-type feature, with transmission electron microscopy (TEM) and atomic absorption spectroscopy (AAS)-quantified Ag loading, and we quantify antibacterial activity against *E. coli* and *S. aureus via* growth curves and MIC. By contrasting our protein-free, cationic H-ND–Ag with literature-reported, protein-stabilized, anionic analogues, we delineate the roles of ND carrier charge, size, and Ag distribution in determining efficacy, and we map how autoclaving influences H-ND colloids *versus* H-ND–Ag composites. This integrated analysis provides design rules for autoclave sterilization-compatible, protein-free H-ND–Ag nanocomposites that retain colloidal and antibacterial function. The novelty of this work lies in the use of positively charged H-NDs as carriers for Ag nanoparticles, where a minimal amount of chitosan is employed as a non-covalent binder to immobilize AgNPs on an otherwise chemically smooth H-ND surface. This approach enables the formation of protein-free, cationic, and autoclave-compatible ND–Ag composites with stable Ag dispersion, while directly comparing two carrier sizes and correlating sterilization-induced surface changes with colloidal and antibacterial performance.

## Materials and methods

### Materials

#### Hydrogenation of NDs

In the preparation of H-NDs, we followed the same procedure reported previously.^16^ Briefly, we used commercially available HPHT monocrystalline nanodiamonds – MSY18 with a size range of 0–0.03 µm and a median size of 18 nm and MSY125 with a size range 0–0.25 µm and a median size of 125 nm (Pureon, Switzerland). First, both ND powders were purified and their surface chemistry homogenized by air annealing treatment at 450 °C for 5 h. Subsequently, NDs were hydrogenated by atmospheric-pressure annealing in hydrogen gas at 800 °C for MSY18 (further labelled as H-ND18) and 1000 °C for MSY125 (further labelled as H-ND125) for 6 h. Water-based colloidal dispersions were prepared by dispersion of 1.0 mg of ND powder in 1.0 mL of deionized water and treated by using a rod-type sonicator (Hielscher, UP200 s) at 120 W for 1 h.

#### H-ND–Ag nanocomposites

In the first step, the H-NDs were coated with a thin chitosan layer. The suspension of H-NDs in deionized H_2_O (1 mg NDs/1 mL) was mixed with a solution of chitosan (0.01% *w*/*w*) in 10 mmol HCl in a 3 : 1 (chitosan solution : ND suspension) ratio. This suspension was stirred at 60 °C for 2 h and then left at room temperature for 2 days. Then, the suspensions were washed with deionized water three times by using centrifugation at 14 500×*g* for 20 min for H-ND18 and 10 min for H-ND125, and finally re-dispersed in deionized water by using the rod-type sonicator for 1 min, yielding chitosan-coated H-ND18 and H-ND125.

In the second step, the AgNPs were formed and anchored to the H-ND–chitosan surface. 5 mL of H-ND–chitosan suspension was stirred at 100 °C for 20 min. Then, a freshly prepared silver nitrate solution was added dropwise into the mixture to reach a final molar concentration of 0.04 mol L^−1^ of AgNO_3_. During the addition of AgNO_3_, the mixture was stirred and kept at 100 °C for 10 min in a dark place. Then 5 mL solution of PEI (PEI MAX, Polysciences, 0.01% *w*/*w*) in water was added, and stirred at 100 °C for another 30 min in the dark. PEI was used as a reducing and stabilizing agent due to its high density of amine groups, which enable coordination with Ag^+^ ions, and controlled nanoparticle formation, while preserving the overall cationic character of chitosan and H-NDs. Formation of AgNPs was accompanied by the appearance of a yellow color due to the plasmonic absorption of AgNPs. Such prepared H-ND–Ag samples were washed with deionized water three times by repeated centrifugation at 14 500×*g* for 20 min for H-ND18–Ag and 10 min for H-ND125–Ag, and finally re-dispersed in deionized water by using the rod-type sonicator for 1 min.

#### Autoclave sterilization process

ND samples were sterilized in an autoclave PS 20 A (Chirana) at a temperature of 120 °C and pressure of 101.5 kPa above atmospheric pressure for 1 h in glass vials. The samples were then stored under dark conditions at 4 °C.

### Methods

Dynamic light scattering (DLS) and *ζ*-potential were measured by using a Zetasizer Nano (Malvern Panalytical) equipped with a helium-neon laser (633 nm); the scattering angle was 173°. The refractive index of bulk diamond (2.4) and the viscosity of pure water (0.89004 mPa s at 25 °C) were used. Every sample was measured three times, and each of the three DLS size measurements consisted of 10 runs lasting 10 s. The size values are given as the *Z*-average, a characteristic size, obtained from the intensity-weighted mean hydrodynamic size. The *ζ*-potential value was calculated as the average of 3 zeta potential values using the Henry equation. Both DLS and zeta potential measurements were performed in a disposable *ζ*-potential measurement cell.

X-ray photoelectron spectroscopy (XPS) measurements were carried out using a Kratos AXIS Supra photoelectron spectrometer equipped with a monochromated Al Kα source (1486.6 eV). Samples were prepared by drop-casting H-ND–Ag samples from a DI colloidal solution onto Au substrates, followed by drying in air. The chamber pressure during measurements was approximately 10^−8^ Torr. The emission angle of 54° (between the incident X-ray beam and the photoelectron analyzer) was fixed for all measurements. High-resolution spectra were acquired with a pass energy of 40 eV, an X-ray power of 150 W, and an energy step of 0.1 eV. Reference samples of bulk Ag (solid bulk) and AgCl (pressed powder) were measured without charge compensation, whereas a flood gun was used to compensate for charging on H-ND–Ag samples. Chemical composition was determined from integrated core-level intensities after Shirley background subtraction, with intensities normalized using the corresponding sensitivity factors implemented in the ESCApe 1.6.1.1234 software (Kratos).

Fourier transform infrared spectroscopy (FTIR) was conducted by using a Bruker Vertex 70v spectrometer equipped with a KBr beam splitter and an N_2_-cooled MCT detector. 50 µL of a suspension was drop-cast onto IR-transparent CaF2 substrates and heated briefly at 60 °C to evaporate the liquid. The measurement chamber was evacuated, ensuring low water vapor and CO_2_ partial pressure. All spectra were measured in a transmission regime. Each spectrum represents an average of 128 scans per spectrum collected in the range of 4000–1000 cm^−1^ at 4 cm^−1^ resolution.

TEM images were acquired by using a Tecnai G2 20 (FEI) TEM with a LaB_6_ cathode at an acceleration voltage of 200 kV. The TEM is equipped with a CCD camera Olympus Veleta. Samples for TEM were inspected on a carbon-coated copper grid, which was immersed in ND colloids before observation.

To quantify the silver content in the nanocomposite suspension, an AAS device (Agilent 280FS) was used, employing a laminar premixed flame generated by burning a mixture of acetylene and air at approximately 2300 °C. The analysis was conducted using a wavelength of 328.1 nm.

#### Bacterial strains and culture conditions

Gram-positive bacterial strain *Staphylococcus aureus* ATCC 6538 and Gram-negative bacterium *Escherichia coli* ATCC 8739 were obtained from the Czech collection of microorganisms (CCM, Czechia). Bacterial suspensions were prepared from pure bacterial cultures grown in Tryptone Soy Broth (TSB, Oxoid Ltd, United Kingdom) and were stored in a mixture of TSB and glycerol (25%) at −80 °C. Before the analysis, bacterial suspensions were inoculated into sterile TSB, incubated for 24 h at 37 °C, and centrifuged (5000×*g*, 2 min). Pellets obtained were resuspended to the desired cell density in TSB and used for antimicrobial tests.

#### Bacterial growth in the presence of ND derivatives

In a sterile microplate (Gama Group, Czechia), H-NDs and H-ND–Ag suspensions were first pipetted to a final concentration of 0.1 g mL^−1^, followed by the addition of a bacterial suspension with an optical density (OD) of 0.5 McF. The plate was then incubated in an Eon™ Microplate Spectrophotometer (BioTek, US) at 37 °C for 24 h with dual-axis mixing for 3 s before each measurement. Absorbance at a wavelength of 620 nm was recorded every 30 min for up to 24 h.

#### Minimal inhibitory concentration

ND colloids in appropriate concentrations (0.2 g mL; 0.1 g mL; 0.05 g mL; 0.025 g mL; 0.02 g mL; 0.01 g mL; 0.005 g mL^−1^) and bacterial suspensions in Tryptic Soy Broth (TSB) adjusted to the OD 0.5 McFarland were added to a sterile 96-well microtiter plate (Gama group, Czechia) in a final volume of 150 µL of fluid in the well. 150 µL of bacterial suspension was used as a control of bacterial growth, and 150 µL of TSB was used as a blank. Before cultivation, absorbance at 620 nm was spectrophotometrically measured (Tecan, Switzerland). The plate was placed on an orbital shaker with an adhesive surface in an incubator (temperature 37 °C, shaker rotation speed 120 min^−1^). After 24 h, absorbance measurement at 620 nm was performed. The difference in measured data before and after the cultivation served as an indicator of bacterial viability in the presence of NDs. MIC was determined as the lowest concentration of NDs, which completely suppressed bacterial growth (*A*_620nm_ < 0.01). The experiments were performed in at least three biological and technical replicates.

#### TEM visualization of bacterial cell interaction with NDs

The bacterial suspension in water was mixed with the colloidal solution of H-ND–Ag and gently stirred using sterile tweezers. The bacteria were then incubated with the nanoparticles under static conditions for 30 min to allow sufficient interaction, although the antibacterial effect of H-ND–Ag had not yet occurred at this stage. Subsequently, the suspension was diluted by transferring 50 µL into 500 µL of dH_2_O to reduce the concentration of the unbound nanocomposite. A TEM grid was immersed in this diluted suspension for 10 min and then left to air dry.

## Results and discussion

### Effect of sterilization on H-ND colloidal stability

**Fig. 1:**
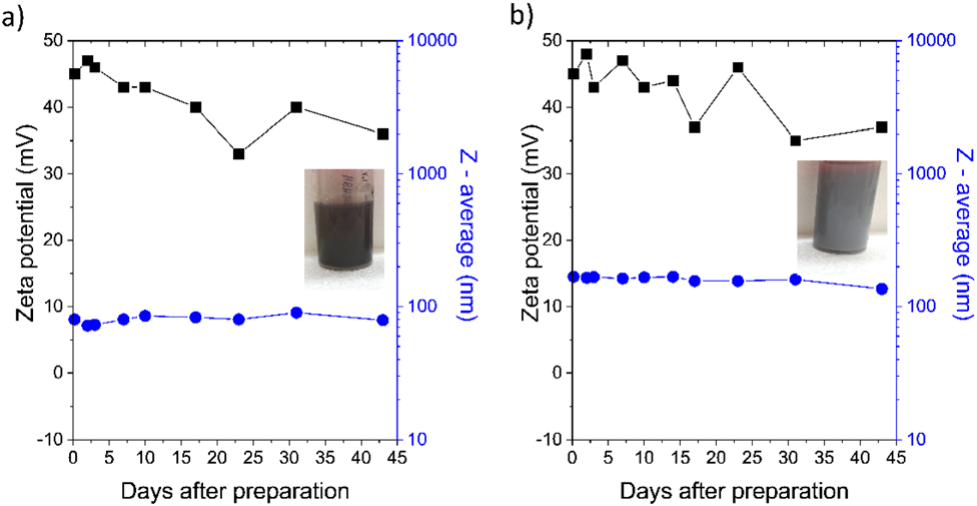
Time dependence of *ζ*-potential (black squares) and *Z*-average particle size (blue circles) for pristine H-ND18 (a) and H-ND125 (b) suspensions at native pH (6–7), supplemented with photos of the corresponding H-ND suspensions.

For all functional materials intended for biological applications and coming into contact with living matter, it is necessary to evaluate how the sterilization process influences their physicochemical properties. We therefore investigated the colloidal stability of both pristine H-NDs and AgNP-decorated H-NDs and suspensions before and after sterilization by autoclaving at 120 °C for 20 min, which is the most commonly used sterilization protocol.^20,21^ Our results showed a significant effect of the autoclave sterilization on the stability of pristine H-ND suspensions in water. Fig. 1 shows the time dependence of *ζ*-potential values and *Z*-average values for H-ND18 (a) and H-ND125 (b) suspensions at their “native” pH (6–7).

Both suspensions initially exhibited high *ζ*-potentials (>40 mV) and nearly stable *Z*-average values over time, consistent with previous reports of good dispersibility and long-term stability.^16,25^ A slow, gradual decrease in *ζ*-potential was observed during storage, although without an immediate loss of stability. Stock samples were stored in the dark in tightly sealed vessels at room temperature. They were opened only to extract the necessary volumes for measurements, which were discarded after measurement.

**Fig. 2:**
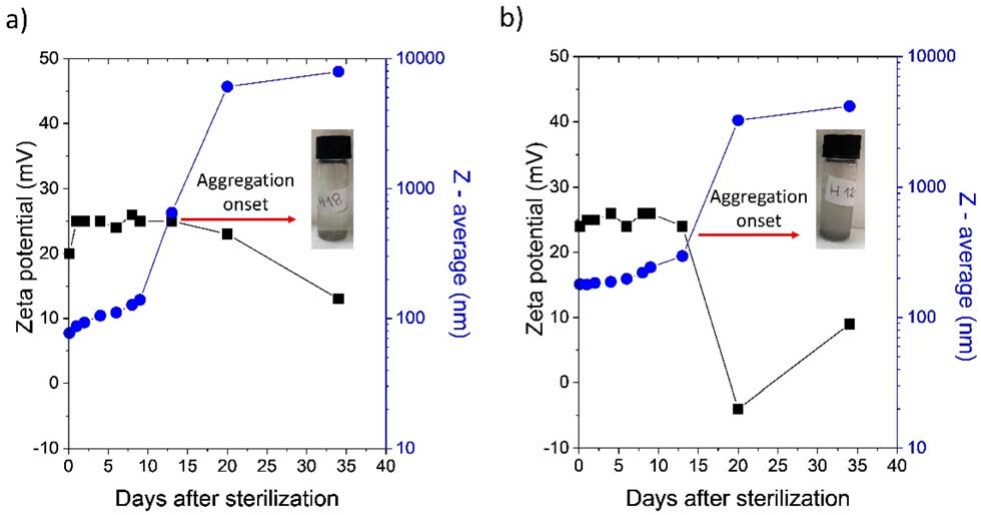
Time dependence of *ζ*-potential and *Z*-average particle size for H-ND18 (a) and H-ND125 (b). The red arrow indicates the onset of aggregation (∼15 days). Insets show the corresponding suspensions after aggregation.

Fig. 2a and b show *ζ*-potential and *Z*-average values for H-ND18 and H-ND125 after sterilization. Immediately after autoclaving, the *ζ*-potential decreased by approximately 20 mV (to ≈25 mV) compared to that of freshly prepared suspensions, yet their colloidal stability was still preserved. The *ζ*-potential was measured immediately after sterilization by autoclaving once the suspension had cooled to room temperature. However, within 20 days, both suspensions underwent aggregation and sedimentation, accompanied by a further decrease in *ζ*-potential and a stepwise increase in *Z*-average. The inverse relationship between *ζ*-potential and *Z*-average size reflects the loss of electrostatic stabilization upon sterilization, leading to particle aggregation as the *ζ*-potential approaches the colloidal stability threshold. This destabilization was consistent across repeated experiments and was visually evident as turbidity and sediment formation (Fig. 2). DLS and *ζ*-potential measurements of destabilized suspensions were already strongly influenced by sedimentation.

We note that samples stored in cool, dark places, with limited air access remained stable for longer, but the overall trend shows that autoclaving markedly compromises the colloidal stability of pristine H-NDs.

**Fig. 3:**
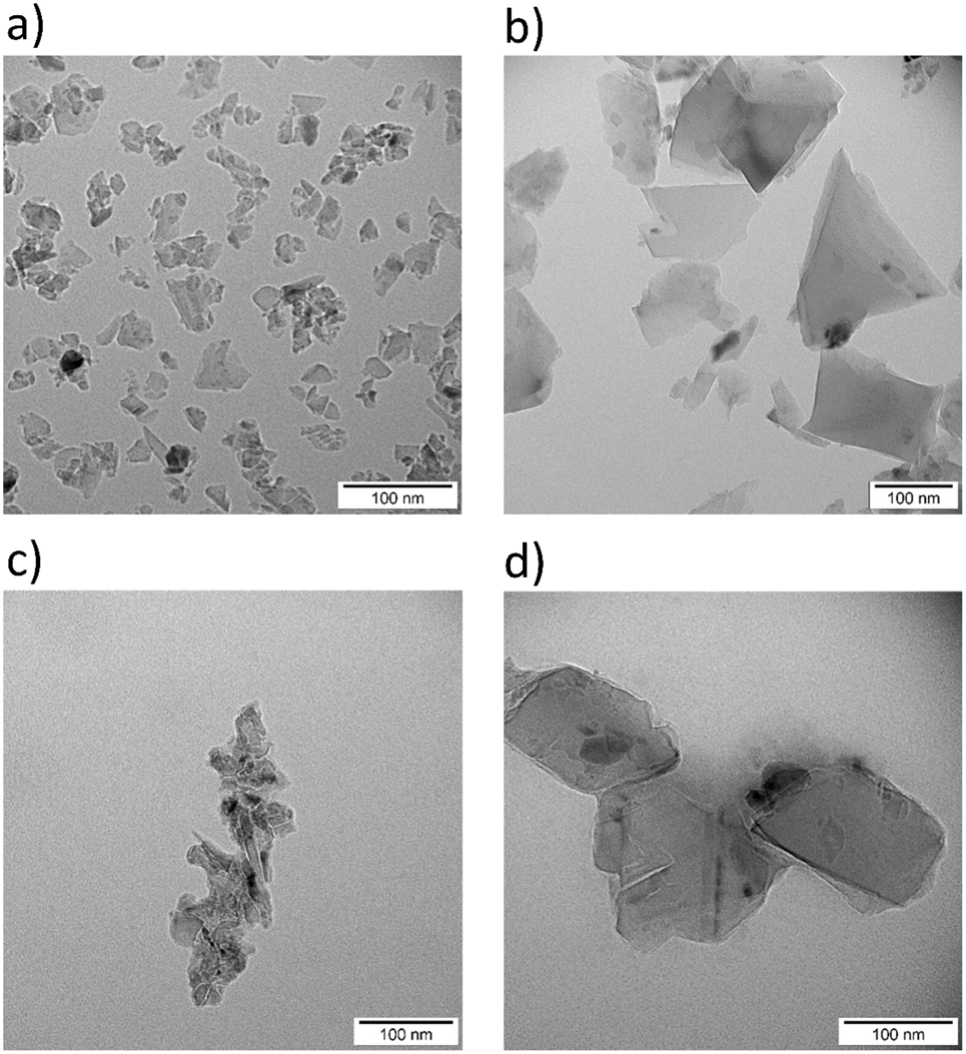
TEM images of H-ND18 before (a) and after autoclave sterilization (b). TEM images of H-ND125 before (c) and after sterilization (d).

TEM images (Fig. 3) support these findings: suspensions not sterilized by autoclaving (Fig. 3a and c) show well-dispersed nanoparticles, whereas autoclaved ones (Fig. 3b and d) display pronounced aggregation.

### H-NDs decorated with Ag nanoparticles and their colloidal stability

**Fig. 4:**
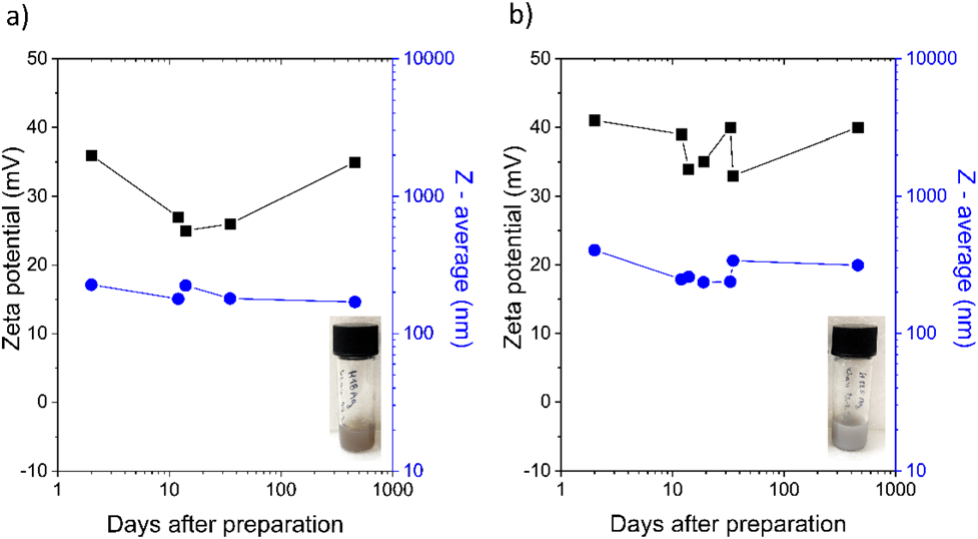
Temporal evolution of *ζ*-potential and *Z*-average for H-ND18–Ag and H-ND125–Ag supplemented with photos of the corresponding H-ND dispersions/suspensions after their preparation. Note the logarithmic scale of the *X* axis.

H-ND18–Ag and H-ND125–Ag nanocomposites were prepared using chitosan, which provided two key functions. First, adsorption of chitosan altered the colloidal stabilization of nanodiamonds, with steric contributions becoming dominant over electrostatic, thus improving dispersibility in aqueous and especially ionic solutions. Second, it facilitated a stable surface for decoration and anchoring of AgNPs upon the addition of a reducing agent. Freshly prepared H-ND18–Ag and H-ND125–Ag suspensions exhibited *ζ*-potentials of 36 and 41 mV, respectively. The long-term temporal evolution of *ζ*-potential and *Z*-average (Fig. 4) shows that both parameters/values remained unchanged for more than one year, with only minor *ζ*-potential fluctuations that did not lead to aggregation. This confirms that steric stabilization dominates the colloidal stability of the suspensions.

PEI was selected as the reductant because of its mild reduction capability and its cationic character. In contrast to our previous study using anionic PVP, which decreased *ζ*-potential significantly,^16^ PEI avoided charge compensation effects. In contrast to pristine H-NDs, the Ag-decorated nanocomposites remained colloidally stable even after autoclaving. We attributed this stability to effective steric stabilization provided by both chitosan and PEI.

**Fig. 5:**
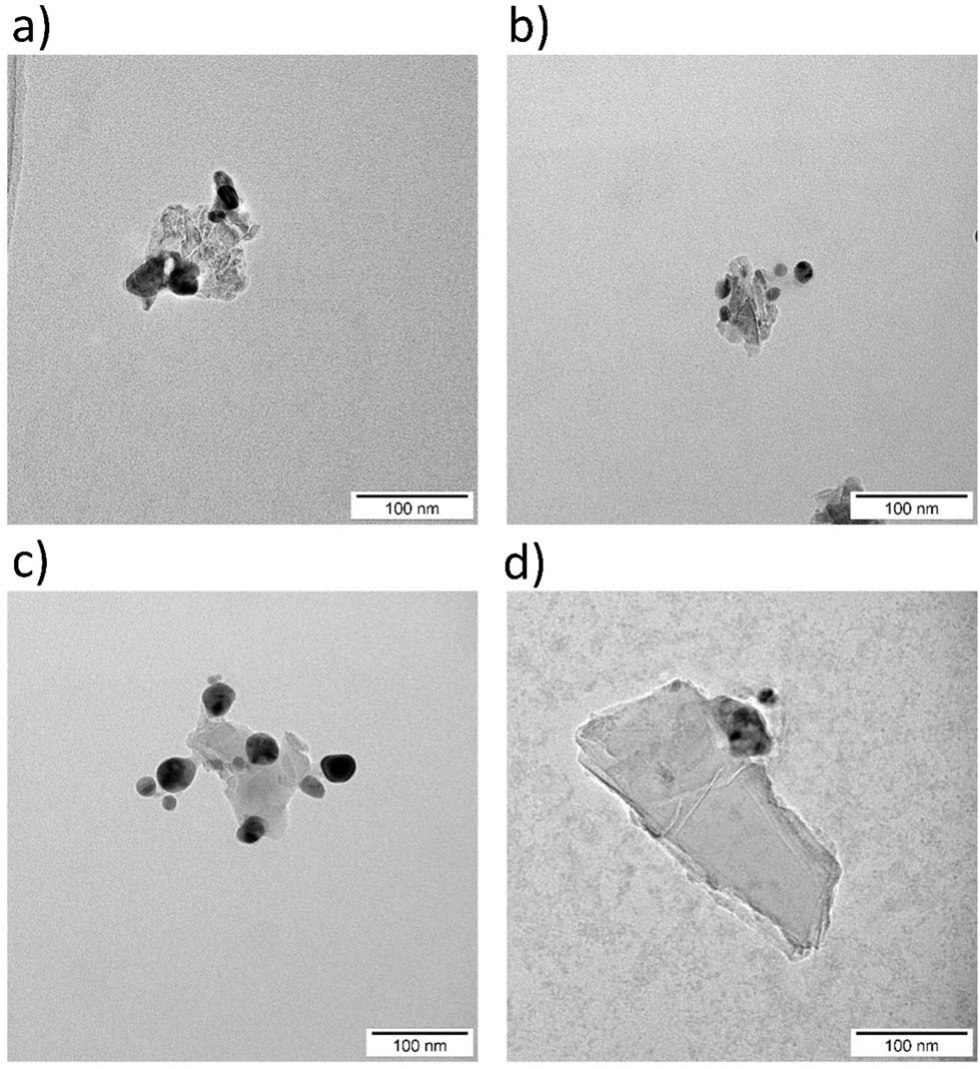
TEM images of H-ND18–Ag nanocomposites, before (a) and after (b) autoclave sterilization, and TEM images of H-ND125–Ag nanocomposites, before (c) and after (d) autoclave sterilization.

DLS data (Fig. 1 and 4) indicate that nanocomposites exhibit larger *Z*-average values than neat H-NDs, partly due to particle enlargement by AgNP decoration and partial aggregation during synthesis. TEM images (Fig. 5) show AgNPs (∼20 nm) anchored variably on the H-ND surface, typically 1–10 AgNPs per nanodiamond. Most often, nanocomposites with 1–2 AgNPs were observed.

**Fig. 6:**
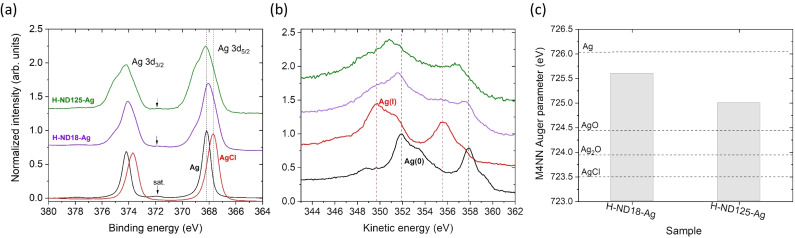
XPS Ag 3d core-level (a) and Ag MNN Auger spectra (b) measured for Ag and AgCl reference samples and H-ND–Ag samples. Auger parameters derived for the H-ND–Ag samples (c). Dashed lines indicate reference values of the Auger parameters.

XPS analysis was employed to further confirm the presence and chemical state of silver in the H-ND–Ag nanocomposites (Fig. 6). Quantitative analysis (Table S1, SI) shows that pristine H-NDs are chemically clean, with only ∼1 at% oxygen, while Ag-decorated samples contain measurable amounts of Ag, together with residual Cl and N originating from the synthesis and surface functionalization.

The Ag concentration is higher for H-ND18–Ag (8.8 at%) than for H-ND125–Ag (2.3 at%), consistent with the larger specific surface area of smaller NDs. Chlorine detected in the samples may be associated both with AgCl-like species and with counterions in the chitosan-based surface layer. Nitrogen is attributed primarily to the chitosan coating and, to a lesser extent, to residual PEI or nitrate species from the synthesis.

The Ag 3d core-level spectra (Fig. 6a) reveal broadened features compared to metallic Ag, indicating the presence of multiple chemical states. The dominant contribution corresponds to metallic Ag, while minor components consistent with AgCl-like species and a mixture of Ag oxides could also be observed. This assignment is supported by Ag MNN Auger analysis (Fig. 6b and c), where the derived Auger parameters are close to those of metallic Ag, particularly for H-ND18–Ag, whereas H-ND125–Ag shows a slightly larger contribution from non-metallic species.

A detailed analysis of the XPS data, including the calibration procedure and full compositional tables, is provided in the SI. Although elemental mapping was not performed, the spatial distribution of AgNPs observed in TEM, together with XPS analysis and AAS quantification (see below), consistently confirms their successful immobilization on the nanodiamond surface.

Noticeably, autoclaving did not significantly alter AgNP size or morphology. Steric stabilization is a well-established concept to prevent nanoparticle aggregation in complex media.^29^ For nanodiamonds, most reported approaches rely on complex covalent functionalization.^30–32^ Our strategy is simpler: non-covalent adsorption of chitosan stabilizes positively charged H-NDs through a combination of electrostatic interactions, van der Waals forces, and possibly hydrophobic contributions.^16^ This non-covalent approach provides excellent stability even after autoclaving and preserves the inherent intrinsic H-ND properties, such as their positive *ζ*-potential.

### Autoclave sterilization effect on H-ND surface chemistry and electronic properties

**Fig. 7:**
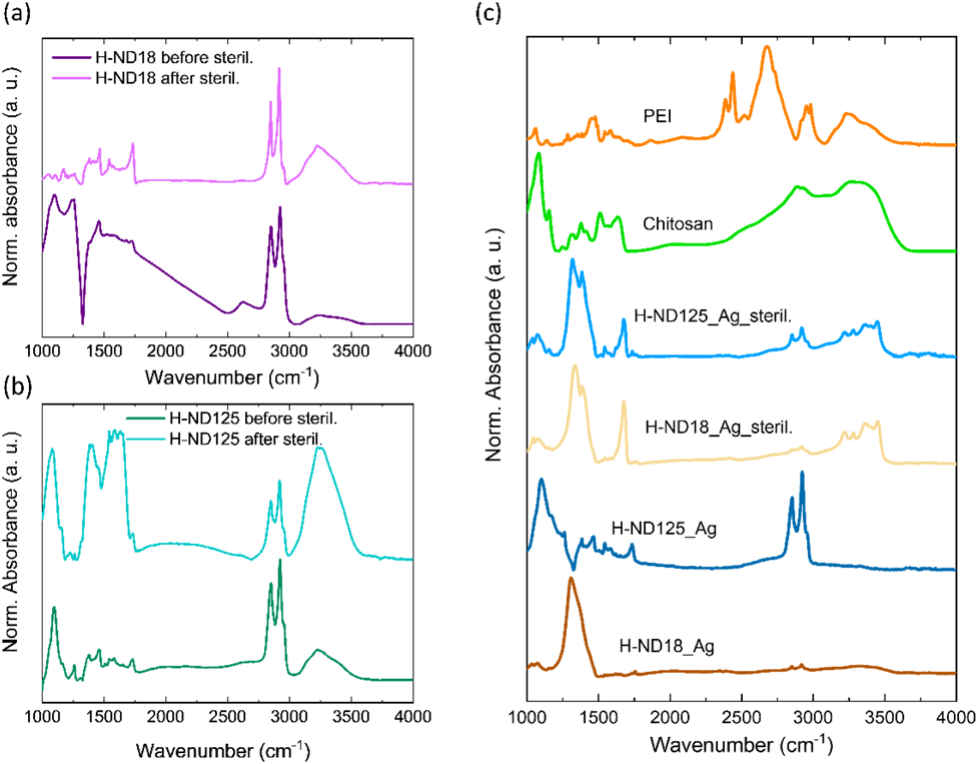
FTIR spectra of H-ND18 (a) and H-ND125 (b) before and after autoclave sterilization. FTIR spectra of H-ND18–Ag and H-ND125–Ag nanocomposites before and after autoclave sterilization, shown together with reference FTIR spectra of chitosan and PEI.

The DLS and TEM data demonstrated that sterilization leads to *ζ*-potential reduction and colloidal destabilization of pristine H-NDs. FTIR analysis provides insight into the underlying surface-chemical and electronic changes responsible for this behavior. Fig. 7a and b show FTIR spectra of H-ND18 and H-ND125 before and after sterilization, respectively. Before sterilization by autoclaving, both samples displayed strong C–H_*x*_ stretching (2800–3000 cm^−1^) and bending modes (1460 cm^−1^), a residual weak C=O band at 1720 cm^−1^, and a broad O–H stretching between 3000 and 3600 cm^−1^. A distinct feature at ∼1330 cm^−1^, particularly pronounced in H-ND18, is attributed to Fano-type destructive interference between the zone-center phonons and free carriers in the near-surface layer,^24^ which is consistent with previously reported charge carrier features/signature in hydrogenated HPHT nanodiamonds.^25^ After sterilization, the 1330 cm^−1^ feature was strongly suppressed, while O–H and C=O bands increased in intensity, indicating partial oxidation of the hydrogenated surface, consistent with earlier studies showing that hydrogenated detonation nanodiamonds begin to oxidize at temperatures as low as 100–150 °C.^26^ For H-ND18, this 1330 cm^−1^ feature was much more intense than for H-ND125, reflecting its higher surface-to-volume ratio; yet it was diminished after sterilization, underlining the sensitivity of near-surface free carriers to oxidative modification. The disappearance of the free-carrier signature confirms that sterilization impacts both near-surface electronic states and surface chemistry.

Beyond these spectral changes, the observed decrease in *ζ*-potential and subsequent colloidal destabilization can be understood in terms of coupled surface-chemical and electronic effects. Hydrogenated nanodiamonds are known to exhibit surface conductivity and charge-transfer behaviour sensitive to adsorbates and the surrounding medium.^16,33^ In this framework, the positive *ζ*-potential does not arise solely from static surface functional groups, but reflects a dynamic interfacial charge state stabilized by hydrogen termination and the near-surface electronic structure.

Autoclave sterilization likely perturbs this balance. The suppression of the ∼1330 cm^−1^ IR feature indicates disruption of near-surface charge carriers, while the increase in O–H and C=O bands points to partial oxidation and reconfiguration of surface adsorbates. These changes can lead to a partial “discharging” of the nanodiamond surface, accompanied by a loss of stabilized interfacial charge and thus a decrease in *ζ*-potential.

In addition, changes observed in the C–H_*x*_ spectral region after sterilization may reflect not only modifications of intrinsic surface termination, but also altered adsorption of adventitious organic species due to modified hydration and surface polarity. Together, these effects provide a consistent explanation for the reduced *ζ*-potential and the gradual loss of colloidal stability observed after autoclaving.

The FTIR spectrum of PEI (Fig. 7c) is dominated by a broad, structured absorption envelope spanning approximately 2200–2800 cm^−1^. This feature arises from N–H stretching overtones and combination bands of protonated secondary amine groups (–NH_2_^+^–), reflecting the hydrochloride salt form of the polymer. Additional bands include C–H stretching modes near 2850–2950 cm^−1^, N–H deformation modes in the 1560–1620 cm^−1^ region, and C–N stretching contributions near 1000–1200 cm^−1^. The prominent ammonium-type absorption between 2200 and 2800 cm^−1^ is characteristic of linear PEI in its protonated form.^34^

The FTIR spectrum of chitosan (Fig. 7c) is characterized by a broad O–H/N–H stretching envelope between 3150 and 3500 cm^−1^, C–H stretching modes near 2870 cm^−1^, a C=O stretching band (amide I) at ∼1650 cm^−1^, N–H bending (amide II) near 1560 cm^−1^, and C–O–C stretching contributions in the 1000–1150 cm^−1^ region, consistent with its partially deacetylated polysaccharide structure.^35^

FTIR spectra of the Ag-decorated nanocomposites (Fig. 7c) also showed strong signals at ∼1330 cm^−1^ before sterilization, though with opposite interference patterns in H-ND18–Ag and H-ND125–Ag. The origin of this difference is unclear but may be related to chitosan or AgNPs influencing transfer-doping, as suggested in our earlier work.^16^ After sterilization by autoclaving, both nanocomposites exhibited more uniform spectra dominated by chitosan-related features, reflecting surface modification. Peaks at 3150–3500 cm^−1^ (N–H_*x*_), 2800–3000 cm^−1^ (C–H_*x*_), 1680 cm^−1^ (C=O), and 1315–1380 cm^−1^ (C–O, C–H_*x*_) confirm strong polymer adsorption. In contrast, pristine hydrogenated nanodiamonds are characterized mainly by C–H_*x*_ stretching and bending modes, with only minor contributions from oxygen-containing groups. The composite spectra thus represent a superposition of nanodiamond and polymer contributions. From the two polymers involved, PEI and chitosan, the spectra obviously reflect more of the spectral features found in the spectrum of chitosan. This agrees with the previously confirmed adhesion of chitosan on the H-ND surface^16^ and also the role of PEI that has been used only as a reductant of Ag^+^ ions, added to the already chitosan-coated H-ND dispersion. Possibly due to the cationic nature of both polymers, PEI was not significantly bound to H-ND18–Ag and H-ND125–Ag surfaces.

### Quantity of silver in the suspension (AAS)

The amount of silver in the H-ND18–Ag and H-ND125–Ag suspensions was determined by AAS both before and after the autoclave sterilization process to evaluate the amount of Ag attached and possible losses after sterilization. The results are summarized in Table 1, showing that all the samples contained 400–500 mg L^−1^ of Ag. As expected, the H-ND18–Ag sample exhibited slightly higher Ag concentration than the H-ND125–Ag sample, which is consistent with the larger surface area of the smaller H-ND18. After sterilization, both suspensions displayed lower concentrations of silver (6.6% loss for H-ND18–Ag and 9.1% for H-ND125–Ag). We attribute this loss to an electrostatic deposition of H-ND–Ag on the surface of the sterilization bottle, which is composed of negatively charged SiO_2_. Since the concentration of H-NDs in H-ND–Ag suspensions was 1 mg mL^−1^, the mass ratio of NDs to AgNPs is approximately 200 : 1. This is consistent with the observed Ag loadings and the typical number of AgNPs per nanodiamond observed in TEM analysis. The mass ratio of NDs to AgNPs was selected based on synthesis conditions that ensured the formation of Ag nanoparticles of comparable size for both carriers and their preferential immobilization on the nanodiamond surface.

**Table 1:** The concentration of silver in the H-ND18–Ag and H-ND125–Ag nanocomposites before (fresh) and after autoclave sterilization (sterile). The concentration of H-NDs in the H-ND–Ag suspensions was 1 mg mL^−1^

Sample	Ag concentration (mg L^−1^)
H-ND18–Ag fresh	498
H-ND18–Ag sterile	465
H-ND125–Ag fresh	471
H-ND125–Ag sterile	428

Taken together, the combined structural and spectroscopic analyses provide consistent evidence for the successful formation of H-ND–Ag nanocomposites. TEM imaging reveals discrete, electron-dense Ag nanoparticles immobilized on nanodiamond surfaces, while AAS quantifies the corresponding silver loading. XPS analysis further confirmed the presence of silver in the nanocomposites. The Ag 3d and Auger spectra indicate predominantly metallic Ag with a partial contribution of AgCl-like species, likely formed due to interaction with residual chloride ions during synthesis. No significant AgO_*x*_ contribution was observed. FTIR spectra reflect the presence of the chitosan binder and associated surface modification, and DLS/*ζ*-potential measurements demonstrate the resulting colloidal stability. Additional Raman data (Fig. S1) show that AgNP decoration induces plasmon-related modifications of the Raman response, further supporting the presence of Ag nanostructures on the nanodiamond surface. While these effects are not analyzed in detail here, they are consistent with the formation of Ag-decorated nanodiamond composites.

Together, these complementary observations confirm the successful immobilization and stabilization of Ag nanoparticles on H-NDs, providing a well-defined material platform for subsequent antibacterial investigations.

### Effect of H-NDs and H-ND–Ag on Gram-positive and Gram-negative bacteria

**Fig. 8:**
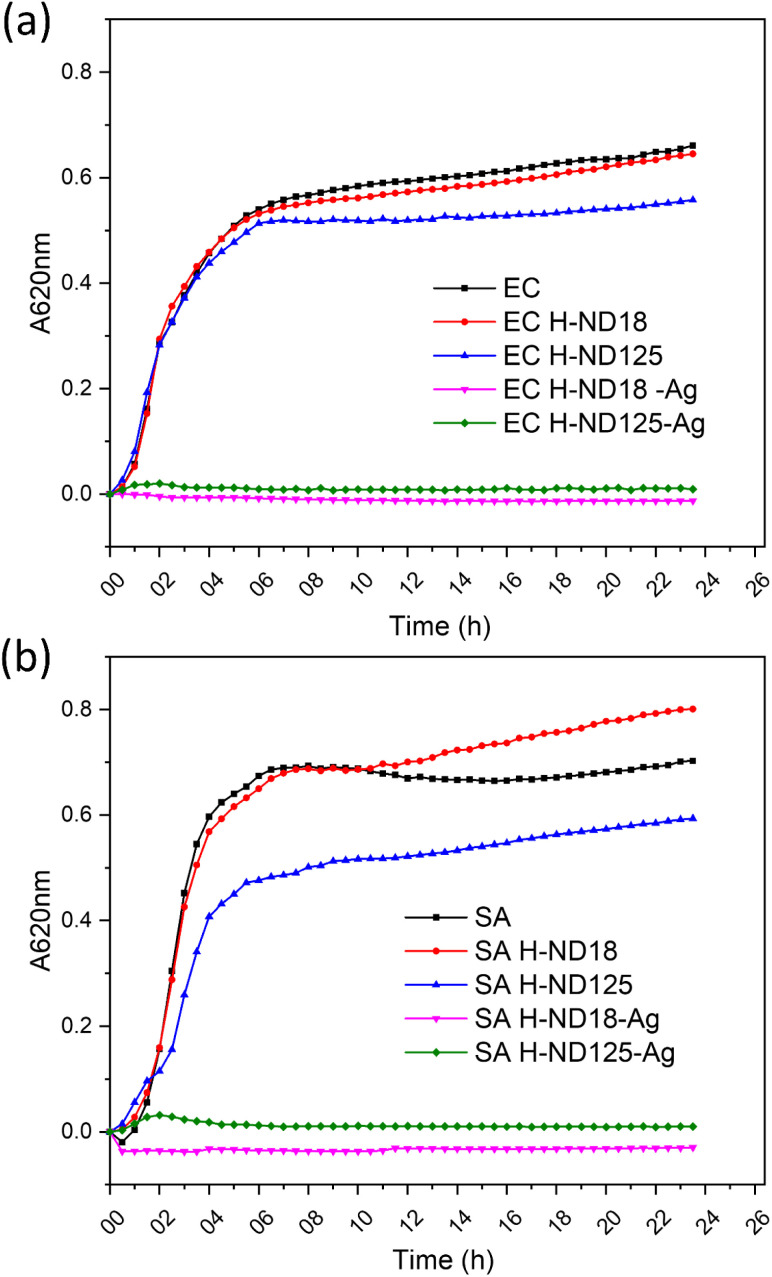
Growth curves of *E. coli* (EC) (a) and *S. aureus* (SA) (b) cultivated in the presence of pristine H-ND18 and H-ND125 and Ag-decorated H-ND18–Ag and H-ND125–Ag nanocomposites.

**Fig. 9:**
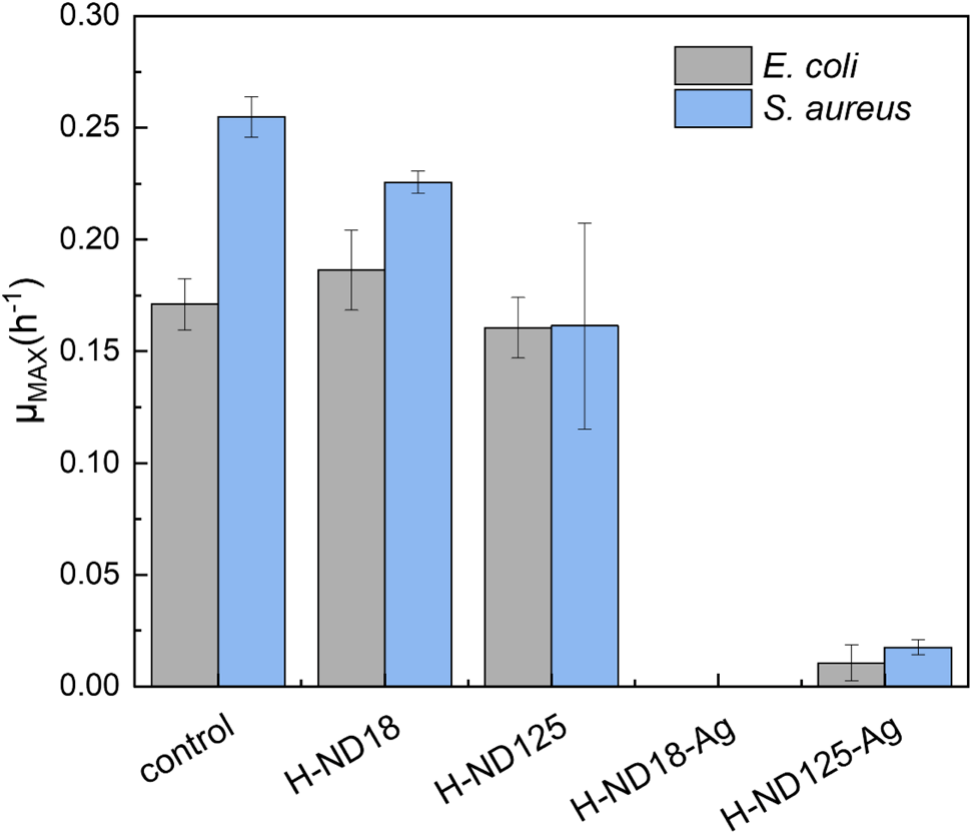
Maximum specific growth rate of *E. coli* and *S. aureus* during the cultivation in the presence of pristine H-ND18 and H-ND125, and H-ND18–Ag and H-ND125–Ag nanocomposites.

The antibacterial performance of the prepared H-ND–Ag composites was evaluated using the representative Gram-positive bacterium *S. aureus* and the Gram-negative bacterium *E. coli*, chosen to reflect differences in the cell wall composition/structure, cell shape, and size, and charge distribution. Growth curves of both bacterial species were recorded in the presence of pristine H-NDs (H-ND18 and H-ND125) and the silver-decorated H-ND–Ag (H-ND18–Ag and H-ND125–Ag). The results plotted in Fig. 8 show a clear difference in antibacterial activity between pristine H-NDs and Ag-decorated H-NDs. Both H-ND18–Ag and H-ND125–Ag exhibited a pronounced inhibitory effect against *S. aureus* and *E. coli* compared to the control without the addition of NDs. In contrast, among the pristine H-NDs, both H-ND18 and H-ND125 induced only mild growth inhibition of *S. aureus*, which is also supported by the differences in their maximum specific growth rates (Fig. 9).

This observation is consistent with the limited studies reporting weak or negligible antibacterial properties of NDs with a considerable amount of surface C–H_*x*_ bonds,^36,37^ yet, to our knowledge, no antibacterial studies on fully H-terminated NDs (whether DND or HPHT) have been reported. We show that H-NDs have at most moderate, medium-dependent antibacterial effects rather than robust bactericidal action. In the clearest assay on detonation NDs, Jira *et al.* used partially hydrogenated DNDs with a mixed C–H_*x*_ and C–O/C=O termination (*i.e.*, only partially hydrogenated surfaces retaining residual oxygen atoms) and observed ∼45% CFU reduction of *E. coli* in Mueller–Hinton, with weaker, transient effects in Luria–Bertani likely tied to protein corona formation and *ζ*-potential changes.^37^

By contrast, deliberately cationized NDs (quaternized or cationic-polymer-grafted) show clear and more consistent bactericidal/anti-biofilm activity across Gram-positive/negative strains and in polymer matrices that is consistent with membrane-active mechanisms and higher surface charge density.^38–41^ Consistent with our results reported here, positively charged H-NDs (fully or partially hydrogenated) behave as milder, context-sensitive inhibitors, whereas purpose-engineered cationic NDs are more potent. Notably, a strong bactericidal effect has also been linked to partially oxidized, negatively charged ND surfaces,^36^ underscoring that surface chemistry and charge identity, not simply “positive *vs.* negative” charge polarity, govern the dominant role.

One of the reasons for the observed weak antibacterial performance of pristine H-NDs might be their instability in ionic solutions and/or the fast/rapid formation of a protein corona around H-ND particles,^42^ which is a known phenomenon occurring in protein-rich media.^36,43,44^ The TSB medium used here for bacterial cultivation in the presence of NDs contains approximately 20 g L^−1^ proteins, which can readily adsorb to nanoparticle surfaces and alter their colloidal stability and biological activity.

In this scenario, the binding of H-NDs to the bacterial cell is based only on electrostatic attraction between the positively charged surface of H-NDs and the negatively charged bacterial cell wall. Thus, the subtle differences in the effect of H-NDs on Gram-positive bacteria and on Gram-negative bacteria can be observed. Gram-positive bacteria such as *S. aureus*, have a high accessible amount of negatively charged teichoic and teichuronic acids in their peptidoglycan layer, and show somewhat greater sensitivity to H-NDs. On the other hand, Gram-negative bacteria possess an outer membrane with lipopolysaccharides (LPSs), which carry a negative charge but also act as a steric and permeability barrier, reducing nanoparticle contact. The existence of this dual role has the capacity to reduce actual contact and uptake of H-NDs, which can result in a comparatively weaker effect on *E. coli*. Based on maximum specific growth rates, it is clear that H-ND125 exhibited a stronger inhibition effect than H-ND18 on *S. aureus*. Although smaller particles display stronger antimicrobial effects than larger ones, primarily due to their larger surface area^45–47^ the static culture experimental conditions (with mixing performed only 3 s before each measurement) likely promoted the faster sedimentation of larger H-ND125 nanoparticles, as modelled by Hinderliter *et al.*^48^ So, accelerated deposition increases the likelihood of direct contact with the bacterial surface, which can mimic stronger apparent antibacterial effects of H-ND-125 which may appear despite expected its lower intrinsic biological activity.

**Fig. 10:**
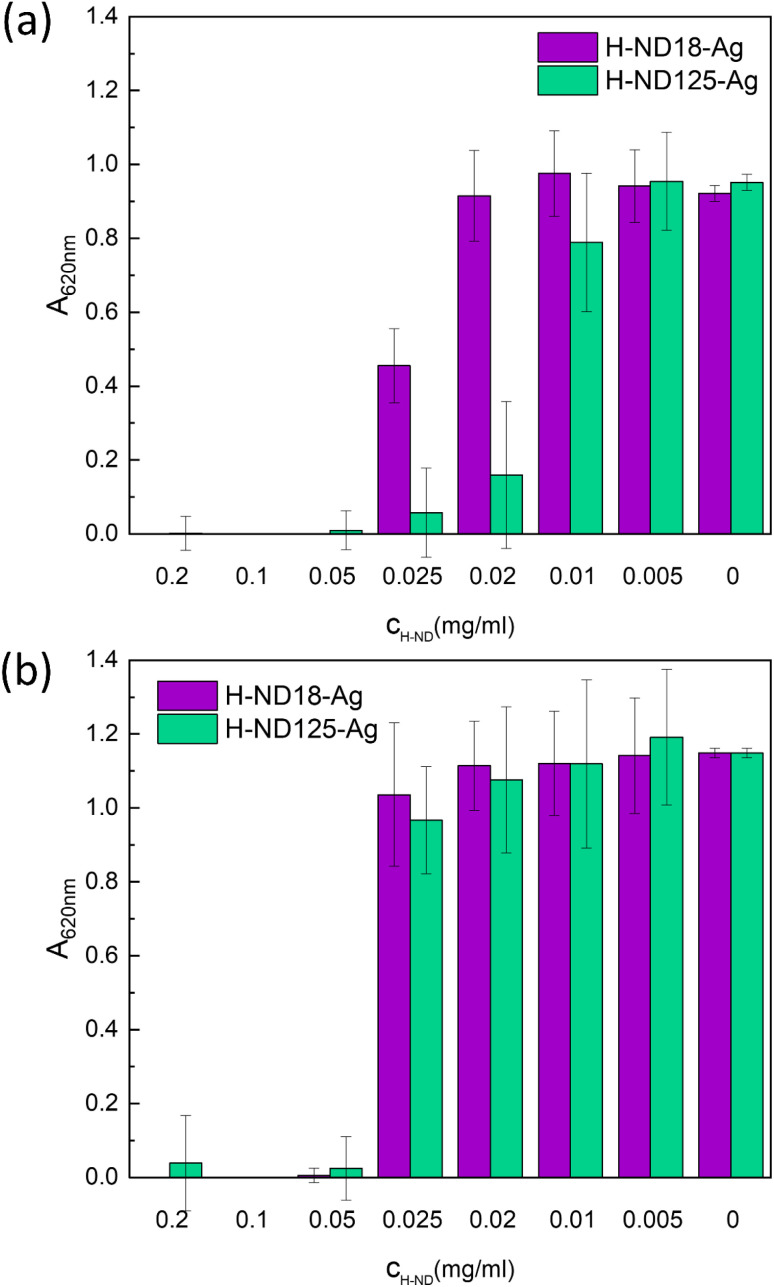
Minimal inhibitory concentration for *E. coli* (a) and *S. aureus* (b) cultivated in the presence of pristine H-ND18 and H-ND125, and Ag-decorated H-ND18–Ag and H-ND125–Ag nanocomposites.

Next, we focused on the minimal inhibitory concentration (MIC) determination of H-ND18–Ag and H-ND125–Ag composites. From all the tested concentrations (0.005 g mL; 0.01 g mL; 0.02 g mL; 0.025 g mL; 0.05 g mL; 0.1 g mL; 0.2 g mL^−1^), complete bacterial inhibition was observed at concentrations of 0.05 g mL^−1^ and higher (Fig. 10) for both *E. coli* and *S. aureus*. In the case of *E. coli* (Fig. 10a), the inhibition of H-ND125–Ag was noticeably higher than for H-ND18–Ag in the 0.01–0.025 mg mL^−1^ concentration range, and the onset of antibacterial activity is not so abrupt. In the case of *S. aureus* (Fig. 10b), both H-ND18–Ag and H-ND125–Ag perform similarly, with a complete inhibition observed from 0.05 mg mL^−1^ and higher.

It can be concluded that both types of prepared composites exhibit significant antibacterial activity, which is dependent on both the concentration and bacterial strain. Given the weak antibacterial activity of pristine H-ND18 and H-ND125 alone, we assume that the pronounced antibacterial activity observed in the composites is primarily attributed to the effect of chitosan and silver, with H-NDs serving as a carrier with a positively charged surface. In this context, NDs do not act as the primary antibacterial agent but provide a platform that promotes interaction with bacterial membranes and enables immobilization and spatial distribution of Ag nanoparticles. Their surface charge can be tuned *via* surface chemistry, allowing control over interfacial interactions and colloidal behavior.^49,50^ In the past, silver was proven to be effective against a wide range of microorganisms,^51^ despite ongoing concerns regarding bacterial resistance.^12,52^ Nevertheless, it appears to be a suitable agent for suppressing pathogens.

Chang *et al.* first demonstrated the use of 100 nm NDs as a carrier for AgNPs^17^ and reported a negative *ζ*-potential of the prepared composites. Similar to our study, both Gram-positive (*S. aureus*) and Gram-negative (*E. coli*) bacteria were investigated so a comparison is relatively straightforward. Compared with Chang *et al.*, who assembled AgNPs on poly-l-arginine-modified, acid-oxidized ∼100 nm NDs and then stabilized the hybrid by BSA adsorption (Ag–ND@BSA, final ζ ≈ −50 mV), our AgNPs immobilized on positively charged hydrogenated NDs (H-ND18–Ag, H-ND125–Ag) were tested without any protein stabilizer.^17^ Chang reported MIC = 15 µg mL^−1^ Ag for *E. coli* (OD_600_, 18 h) and MBC = 31 µg mL^−1^, with sustained kill at 250 µg mL^−1^. Converting our data to Ag-equivalents, *E. coli* MICs were ∼15.9 µg mL^−1^ for H-ND18–Ag and ∼7.5 µg mL^−1^ for H-ND125–Ag; for *S. aureus* both composites gave ∼15–16 µg mL^−1^. Thus, despite lacking BSA (and having a net positive charge), our MICs are comparable to Chang's, and for *E. coli* with the larger H-ND carrier, lower by ∼2×. Together with Chang's observation that their most active hybrid was net negative due to BSA, these results are consistent with a local Ag-patch/dispersion effect (*i.e.*, localized Ag-rich domains acting as contact-active sites), in which carrier size and Ag distribution likely play a dominant role in determining antibacterial efficacy, while surface charge primarily facilitates initial contact.^17^ However, we note that this interpretation is based on indirect structural and biological evidence, and the detailed mechanistic pathways (*e.g.*, contribution of Ag^+^ release, membrane disruption, or ROS generation) were not directly probed in this study. Interestingly, the MIC results revealed that the Gram-positive bacterium *S. aureus* was less susceptible to both H-ND18–Ag and H-ND125–Ag, requiring higher concentrations for growth inhibition compared to *E. coli*, which contrasts with the behaviour observed for pristine H-NDs. In this scenario, multiple factors probably contributed to these outcomes. In this case, chitosan is also cationic and can enhance the binding to the negatively charged cell wall of bacteria.^53^ Chitosan, present in the form of a thin shell on the H-ND surface, may also contribute to the antibacterial properties through its own cationic and membrane–interactive properties.^16^ The antibacterial effect of chitosan^54^ is primarily attributed to ionic and hydrophobic interactions that cause structural damage and increased membrane permeability. Gram-negative bacteria tend to be more sensitive due to their higher hydrophilicity and charge density, which enhances the membrane disruption. Moreover, silver ions have stronger effects on Gram-negative bacteria compared to Gram-positive bacteria, as documented in several studies.^55–57^ This synergistic effect of chitosan and Ag^+^ ions may enhance the destabilization of the membrane together with the disruption of multiple intracellular targets, particularly in *E*. *coli*, compared to *S. aureus.*

**Fig. 11:**
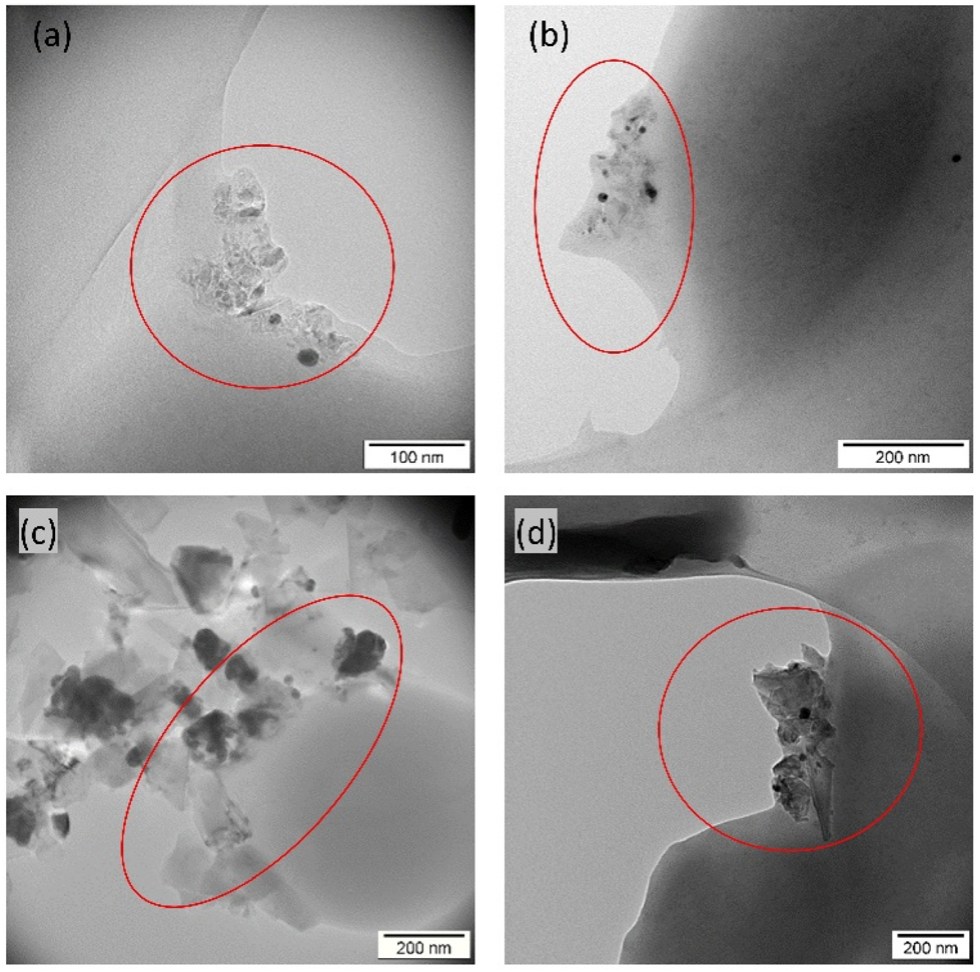
TEM image showing interactions between H-ND–Ag nanocomposites and bacterial cells: H-ND18–Ag in contact with the cell wall of *S. aureus* (a) and *E. coli* (b). and H-ND125–Ag in contact with the cell wall of *S. aureus* (c) and *E. coli* (d). The ovals indicate localized accumulation of H-ND–Ag nanocomposites at the bacterial cell surface, consistent with a contact-mediated (“Ag patch”) antibacterial mechanism.

To better understand the local interactions between the nanocomposites and bacterial cells, TEM analysis was performed after 30 min of incubation. This time was selected based on the growth curve dynamics (Fig. 8) and preliminary TEM observations: after 30 min of exposure, the nanocomposites were already interacting with the bacterial cell walls, but cell death had not yet occurred; in contrast, after 3 hours of contact, intact bacteria were no longer observed on the TEM grid and only cellular fragments were observed (data not shown). Fig. 11a shows three *S. aureus* cocci interacting with H-ND18–Ag. The cell walls appear intact, suggesting that significant structural damage has not yet occurred at this early time point. Small dark aggregates on the bacterial surface indicate initial attachment. The surrounding medium appears relatively homogeneous, with no evident signs of cellular lysis, although localized accumulation of AgNP and ND aggregates at the bacterial cell is visible. The bacterial cell wall reveals some irregularities, possibly due to structural damage induced by AgNPs. Fig. 11b depicts clusters of H-ND18–Ag in contact with the cell wall of *E. coli*. Here, the outer membrane of *E. coli* appears partially damaged. Fig. 11c shows H-ND125–Ag nanocomposites in contact with the cell wall of *S. aureus.* The nanodiamonds exhibit their characteristic angular morphology with sharp-edged formations. Darker regions again indicate AgNPs immobilized on the ND surface. The presence of structural irregularities or possible damage in the *S. aureus* cell wall suggests that H-ND125–Ag interaction may compromise bacterial integrity. As shown in Fig. 11d, the interaction between *E. coli* and H-ND125–Ag is shown, revealing the structural damage, indicating membrane disruption.

It should be noted that the nanodiamond size itself is not the primary determinant of antibacterial activity in this system, as H-NDs act mainly as carriers for AgNPs. While smaller nanoparticles are often reported to exhibit higher intrinsic antibacterial activity, our results indicate that larger H-ND125 carriers remain highly effective when decorated with AgNPs. This can be attributed to their ability to support stable AgNP immobilization and to facilitate contact with bacterial cells, potentially enhanced by sedimentation and increased contact area. These observations highlight that, in carrier-based systems, particle size must be considered in the context of the overall composite structure and delivery mechanism rather than as an isolated parameter.

From an environmental perspective, the long-term fate of H-ND–Ag composites is expected to be governed by the different stabilities of their individual components. AgNPs are known to undergo gradual oxidation and dissolution, leading to the release of Ag^+^ ions, while chitosan and PEI coatings are biodegradable or susceptible to environmental degradation processes. In contrast, relatively chemically inert NDs are generally considered non-biodegradable under environmental conditions. Therefore, over extended time scales, the composite is expected to evolve toward dispersed ND residues with reduced silver content. The overall stability and transformation rate of the system are also expected to depend on environmental conditions, such as light exposure, temperature, and the surrounding medium, which may accelerate degradation processes compared to controlled laboratory storage. While nanodiamonds themselves are widely regarded as biocompatible and having low toxicity,^58^ the environmental impact of released silver species and long-term nanoparticle persistence remains an important consideration.^59^ A detailed assessment of environmental behaviour and biodegradation pathways is beyond the scope of the present study and warrants further investigation.

From an application perspective, the combination of long-term colloidal stability, persistent positive surface charge, and sustained antibacterial activity makes these H-ND–Ag composites particularly suitable for use in durable antimicrobial systems. In contrast to biodegradable materials, their structural and colloidal stability may be advantageous in applications requiring long-term performance, such as antimicrobial surface coatings, filtration membranes, or packaging materials where prolonged resistance to microbial contamination is desired. In this context, the non-degradable nature of nanodiamonds can be considered beneficial, provided that their use is confined to controlled environments.

## Conclusions

This study investigates the impact of steam sterilization on the surface and colloidal stability of hydrogenated HPHT nanodiamonds (H-NDs), their subsequent modification into AgNP-bearing H-ND nanocomposites, and the resulting antibacterial activity against Gram-negative *E*. *coli* and Gram-positive *S*. *aureus*. We demonstrate that positively charged H-NDs serve as effective carriers for AgNPs, yielding long-term stable, autoclave-resilient dispersions when protected by a minimal, non-covalent chitosan shell. Pristine H-NDs alone exhibit only mild and medium-dependent antibacterial activity. The H-ND–Ag composites achieve MIC values of ∼0.025–0.05 mg mL^−1^ (≈7.5–16 µg mL^−1^ Ag), with H-ND125–Ag outperforming H-ND18–Ag against *E. coli*.

FTIR and colloidal measurements indicate that steam sterilization induces surface modifications of pristine H-NDs (surface chemistry and electronic properties), suppressing the diamond-associated ∼1330 cm^−1^ feature and increasing O–H/C=O bands, consistent with surface changes (partial surface oxidation and/or desorption). This was accompanied by a decrease in the positive *ζ*-potential and gradual aggregation upon storage. By contrast, the chitosan/AgNP shell maintained a positive *ζ*-potential and narrow hydrodynamic size distributions (DLS), and provides strong Ag retention, sustaining colloidal stability and antibacterial performance.

Our findings, together with the literature reference values, suggest that antibacterial efficacy is primarily influenced by nanodiamond carrier size, Ag content/distribution, and steric stabilization, while the positive surface charge of H-NDs facilitates initial bacterial contact. This work establishes a protein-free, autoclave-compatible strategy/approach for preparing antibacterial nanodiamond–silver nanocomposites, and provides design guidelines for future optimization of Ag dose, polymer-shell architecture, and medium conditions toward strain-selective or application-specific performance.

## Author contributions

Katerina Kolarova: conceptualization, investigation, formal analysis, data curation, interpretation, writing – original draft, writing – review and editing. Hana Stiborova: formal analysis, data curation, interpretation, writing – review and editing. Simona Lencova: investigation, formal analysis, data curation, visualization, writing – original draft. Maksym Bilozerskyi: investigation, formal analysis. Oleksandr Romanyuk: investigation, formal analysis, data curation, visualization, writing – original draft. Alexander Kromka: supervision, validation, funding acquisition, project administration, writing – review and editing. Stepan Stehlik: conceptualization, funding acquisition, methodology, project administration, supervision, visualization, writing – original draft, writing – review and editing.

## Conflicts of interest

There are no conflicts to declare.

## Supplementary Material

NA-008-D6NA00095A-s001

## Data Availability

The data that support the findings of this study are openly available in Zenodo at https://doi.org/10.5281/zenodo.18503631. Supplementary information (SI) is available. See DOI: https://doi.org/10.1039/d6na00095a.
