# Supplementary material for: Steam-sterilizable cationic nanodiamond–silver composites with enhanced antibacterial activity
Source: Nanoscale Adv. 2026 May 29;8(12):3627–39. doi: 10.1039/d6na00095a (PMC13220093; doi:10.1039/d6na00095a)
Supplement: NA-008-D6NA00095A-s001 [file NA-008-D6NA00095A-s001.pdf]

Supplementary Information (SI) for Nanoscale Advances.
This journal is © The Royal Society of Chemistry 2026

Supporting information for:

Steam-sterilizable cationic nanodiamond–silver composites with enhanced antibacterial activity

Katerina Kolarova[a], Hana Stiborova[b], Simona Lencova[b], Maksym Bilozerskyi[b], Oleksandr Romanyuk[a], Alexander Kromka[a], Stepan Stehlik[a*]

[a] FZU - Institute of Physics of the Czech Academy of Sciences, Cukrovarnicka 10, 162 00 Prague, Czech Republic
[b] Department of Biochemistry and Microbiology, University of Chemistry and Technology, Technicka 5, 166 28 Prague, Czech Republic
* stehlik@fzu.cz

Raman spectra

Raman spectroscopy (457 nm excitation) was used to probe structural changes upon Ag nanoparticle decoration. All samples exhibit the characteristic diamond peak (~1332 cm^{-1}), confirming the preservation of the nanodiamond core after functionalization. Pristine samples H-ND18 and H-ND125 show a mild content of the surface sp2-C, which is typically formed during the thermal hydrogenation process[1].

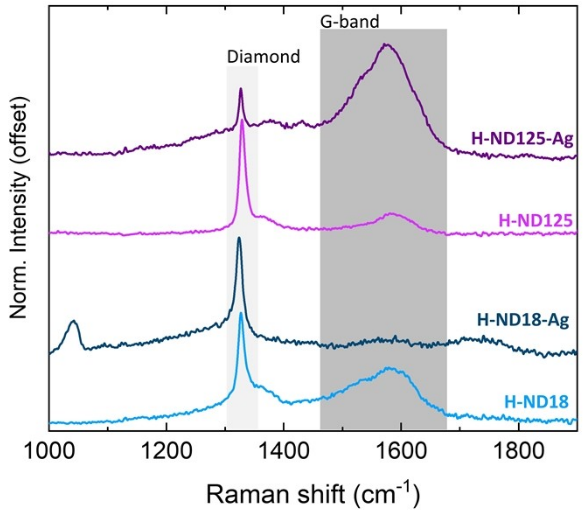

Figure S1. Raman spectra of pristine H-ND18 and H-ND125 and their Ag-decorated counterparts (H-ND18-Ag and H-ND125-Ag). The characteristic diamond peak (~1332 cm^{-1}) is preserved in all samples, while variations in the G-band (~1580 cm^{-1}) indicate changes in the relative contribution of sp^2 carbon upon AgNP decoration.

For H-ND18-Ag, the spectrum is dominated by a sharp diamond peak with a strongly suppressed G-band contribution. In contrast, H-ND125-Ag shows a relatively enhanced G-band compared to its pristine counterpart.
These differences are tentatively attributed to plasmonic effects associated with Ag NPs. For smaller nanodiamonds (H-ND18), the higher surface curvature and closer spatial arrangement of AgNPs may promote the formation of localized electromagnetic "hot spots", potentially enhancing the diamond-related signal or suppressing the contribution of sp^2 carbon. In contrast, for larger nanodiamonds (H-ND125), AgNPs are more spatially distributed over extended facets, which may lead to a different

plasmonic response, including enhanced sensitivity to surface sp^2 species and possible local surface modification. We note that this interpretation remains speculative, as a detailed investigation of plasmonic effects is beyond the scope of the present study.

UV-Vis spectra

UV–Vis absorption spectroscopy was used as a supplementary technique to probe the presence of Ag nanoparticles. For the H-ND18 system, the relatively transparent colloidal suspension allows reliable measurement, and a weak plasmonic feature associated with AgNPs can be identified at ≈ 420 nm. In contrast, the H-ND125 suspension is strongly turbid due to the larger particle size, and the optical response is dominated by light scattering. Under these conditions, the plasmonic signal of AgNPs cannot be reliably resolved.
Therefore, UV–Vis spectroscopy provides only limited and size-dependent information in this system. The presence and immobilization of Ag nanoparticles are primarily confirmed by complementary techniques (TEM, AAS, and XPS).

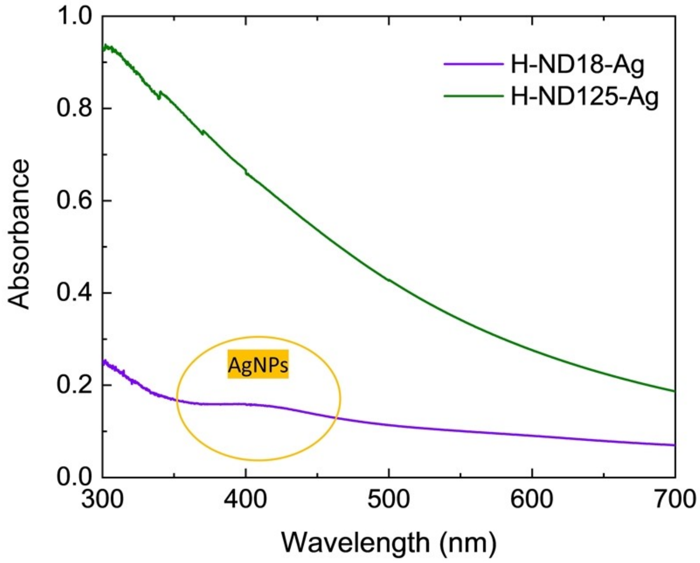

Figure S2. UV-Vis absorption spectra of H-ND18-Ag and H-ND125-Ag samples.

XPS analysis

Atomic concentrations of H-ND18–Ag and H-ND125–Ag samples were determined by XPS (Table S1). The pristine H-ND samples show predominantly carbon (~99 at.%) with ~1 at.% oxygen, confirming effective purification and hydrogenation.

Table S1 | *Atomic concentrations of H-ND, H-ND–Ag nanocomposites, and Ag and AgCl reference samples determined by XPS. The presence of Cl and N residues was confirmed in the H-ND–Ag samples, and the Ag/Cl ratio indicates that Ag is predominantly present in metallic form.*

SAMPLE	Concentration, at.%					
	C	O	N	Ag	Cl	$Au_{subst.}$
H-ND18	98.9	1.1	0.0	0.0	0.0	0.0
H-ND125	98.6	1.3	0.0	0.0	0.0	0.1

							Ag/Cl
**H-ND18-Ag**	73.1	7.4	4.0	8.8	4.7	2.0	1.9
**H-ND125-Ag**	88.0	2.3	1.0	2.3	1.5	4.9	1.5

**References**	**C**	**O**	**N**	**Ag**	**Cl**	**Na**	**Ag/Cl**
**O(C)/Ag**	28.5	2.1	-	69.4	-	-	-
**O(C)/AgCl**	37.6	10.1	-	24.1	24.3	3.9	1.0

In the Ag-decorated samples, silver concentrations of 8.8 at.% (H-ND18–Ag) and 2.3 at.% (H-ND125–Ag) were measured. The higher Ag content observed for smaller nanodiamonds is consistent with their larger specific surface area.
In addition to Ag, Cl and N were detected in both samples. Chlorine may originate from AgCl-like species as well as from counterions associated with the chitosan-based surface layer. Nitrogen is attributed primarily to the chitosan coating and, to a lesser extent, to residual PEI or nitrate species from the synthesis.
The Ag/Cl ratio exceeds unity for both samples (1.9 for H-ND18–Ag and 1.5 for H-ND125–Ag), indicating that silver is predominantly present in a non-chloride form. For comparison, Ag and AgCl reference samples were measured under identical conditions. Carbon and oxygen detected on reference surfaces are attributed to adventitious contamination, while sodium residues were observed in the AgCl reference. As expected, the AgCl reference exhibits an Ag/Cl ratio close to unity.

Calibration of XPS spectra

The XPS spectra were calibrated using reference measurements of metallic Ag (bulk) and pressed AgCl powder. Figure S3 includes spectra of these references together with two representative nanocomposite samples: H-ND18–Ag (discussed in the main text) and an additional, Cl-enriched H-ND18–Ag sample.

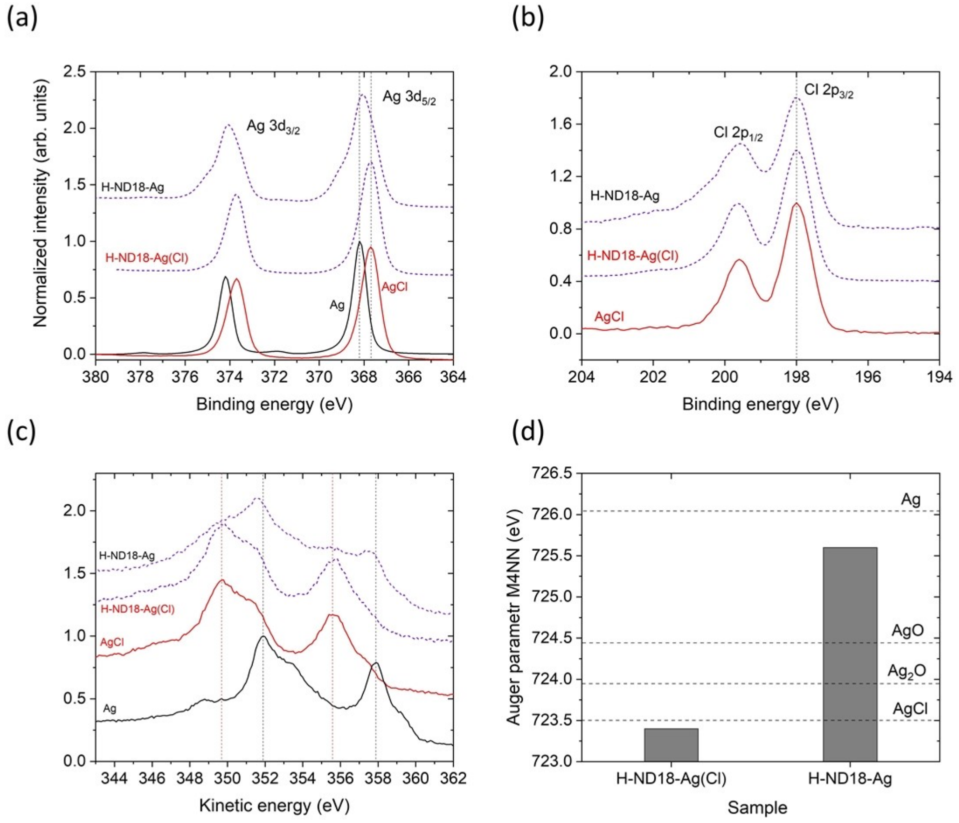

Figure S3. XPS core-level spectra of (a) Ag 3d, (b) Cl 2p, (c) Ag MNN Auger transitions, and (d) derived Auger parameters. Spectra of Ag and AgCl reference samples are shown as measured. Spectra of H-ND–Ag and Cl-enriched H-ND–Ag samples were calibrated using the Cl 2p position of the AgCl reference. The Auger parameters confirm the presence of both metallic Ag and AgCl-like species in the nanocomposites.

The Cl-enriched sample, exhibiting a higher relative Cl content, was used to facilitate reliable energy calibration based on the well-defined Cl 2p peak of AgCl (198.0 eV). Spectra of the nanoparticle samples were aligned to this reference position, resulting in consistent C 1s peak positions within 285 ± 0.3 eV[2]. The Cl-enriched sample shows higher Ag and Cl concentrations (11.1 at.% and 10.7 at.%, respectively), with an Ag/Cl ratio close to unity, consistent with a dominant AgCl-like character. This is reflected in both the Ag 3d core-level spectra and the Ag MNN Auger transitions, which closely resemble those of the AgCl reference. In contrast, the samples discussed in the main text exhibit lower Cl content and a dominant metallic Ag contribution.

The measured Auger parameters further support this interpretation. The Cl-enriched sample yields a value characteristic of AgCl (~723.4 eV), whereas the samples presented in the main text show higher values closer to metallic Ag.

The binding energy difference between O 1s and Ag $3d_{5/2}$ was approximately 164.7 eV for all nanocomposite samples and the AgCl reference. Reported values for Ag oxides are significantly lower[3], indicating that fully oxidized Ag species are not the dominant component.

References

1 A.-I.- Ahmed, S. Mandal, L. Gines, O. A. Williams and C.-L. Cheng, *Carbon*, 2016, **110**, 438–442.
2 O. Romanyuk, Š. Stehlík, J. Zemek, K. Aubrechtová Dragounová and A. Kromka, *Nanomaterials*, 2024, **14**, 590.
3 T. C. Kaspar, T. Droubay, S. A. Chambers and P. S. Bagus, *J. Phys. Chem. C*, 2010, **114**, 21562–21571.
